# 2-Butanone as a carbon dioxide mimic in attractant blends for the Afrotropical malaria mosquitoes *Anopheles gambiae* and *Anopheles funestus*

**DOI:** 10.1186/s12936-017-1998-2

**Published:** 2017-08-24

**Authors:** Monicah M. Mburu, Collins K. Mweresa, Philemon Omusula, Alexandra Hiscox, Willem Takken, Wolfgang R. Mukabana

**Affiliations:** 1 International Centre of Insect Physiology and Ecology, P.O. Box 30772, Nairobi, 00100 Kenya; 2 School of Biological Sciences, University of Nairobi, P.O. Box 30197, Nairobi, 00100 Kenya; 3 Laboratory of Entomology, Wageningen University and Research, P.O. Box 16, 6700 AA Wageningen, The Netherlands; 4 School of Biological and Physical Sciences, Jaramogi Oginga Odinga University of Science and Technology, P.O. Box 210, Bondo, 40601 Kenya; 5 Science for Health, P.O. Box 44970, Nairobi, 00100 Kenya; 6 International Centre for Aids Care and Treatment Program, Ministry of Health, Jaramogi Oginga Odinga Teaching and Referral Hospital, P.O. Box 849, Kisumu, 50100 Kenya

## Abstract

**Background:**

Most odour baits designed to attract host-seeking mosquitoes contain carbon dioxide (CO_2_), which enhances trap catches, given its role as a mosquito flight activator. However, the use of CO_2_ is expensive and logistically demanding for prolonged area-wide use.

**Methods:**

This study explored the possibility of replacing organically-produced CO_2_ with 2-butanone in odour blends targeting host-seeking malaria mosquitoes. During semi-field and field experiments MM-X traps were baited with a human odour mimic (MB5 blend) plus CO_2_ or 2-butanone at varying concentrations. Unbaited traps formed a control. The attraction of *Anopheles gambiae s.s.*, *Anopheles arabiensis* and *Anopheles funestus* to these differently baited traps was measured and mean catch sizes were compared to determine whether 2-butanone could form a viable replacement for CO_2_ for these target species.

**Results:**

Under semi-field conditions significantly more female *An. gambiae* mosquitoes were attracted to a reference attractant blend (MB5 + CO_2_) compared to MB5 without CO_2_ (P < 0.001), CO_2_ alone (P < 0.001), or a trap without a bait (P < 0.001). Whereas MB5 + CO_2_ attracted significantly more mosquitoes than its variants containing MB5 plus different dilutions of 2-butanone (P = 0.001), the pure form (99.5%) and the 1.0% dilution of 2-butanone gave promising results. In the field mean indoor catches of wild female *An. gambiae s.l.* in traps containing MB5 + CO_2_ (5.07 ± 1.01) and MB5 + 99.5% 2-butanone (3.10 ± 0.65) did not differ significantly (P = 0.09). The mean indoor catches of wild female *An. funestus* attracted to traps containing MB5 + CO_2_ (3.87 ± 0.79) and MB5 + 99.5% 2-butanone (3.37 ± 0.70) were also similar (P = 0.635). Likewise, the mean outdoor catches of *An*. *gambiae* and *An*. *funestus* associated with MB5 + CO_2_ (1.63 ± 0.38 and 0.53 ± 0.17, respectively) and MB5 + 99.5% 2-butanone (1.33 ± 0.32 and 0.40 ± 0.14, respectively) were not significantly different (P = 0.544 and P = 0.533, respectively).

**Conclusion:**

These results demonstrate that 2-butanone can serve as a good replacement for CO_2_ in synthetic blends of attractants designed to attract host-seeking *An. gambiae s.l.* and *An. funestus* mosquitoes. This development underscores the possibility of using odour-baited traps (OBTs) for monitoring and surveillance as well as control of malaria vectors and potentially other mosquito species.

## Background

Malaria vectors require a blood meal to develop their eggs [1] and the process of finding blood hosts is primarily mediated by host odour [2–5]. Carbon dioxide (CO_2)_ is one of the important components of human host odour affecting mosquito host-seeking behaviour [6]. It is thought that this gas activates mosquitoes by eliciting take-off behaviour. The presence of CO_2_ then sustains the mosquitoes in host-seeking flight [6, 7], guiding them towards their blood meal hosts [3]. It is not surprising, therefore, that CO_2_ is a key ingredient of synthetic mosquito attractants for host-seeking mosquitoes [8]. The application of this gas from pressurized cylinders, fermenting sugar (i.e., sucrose) or molasses and/or the use of dry ice present major challenges to the use of CO_2 -_based mosquito attractants under field conditions. The gas cylinders are heavy, bulky, expensive and prone to leakages [9] and dry ice can be difficult to obtain, transport and store [9–12]. Whilst CO_2_ produced by fermenting refined sugar or molasses can offer a solution to these problems [13] this method of CO_2_ production is also expensive and presents logistical challenges when used on a large scale because the gas is only produced over one trapping night (ca ten hours) and must be replenished daily.

In a study by Turner et al. 2-butanone was identified as a potential replacement for CO_2_ in a synthetic blend of mosquito attractants [14]. These authors demonstrated the capacity of 2-butanone to induce a dose-dependent activation of the cleavage product A (cpA) CO_2_ receptor neuron in the maxillary palps of *Anopheles gambiae, Aedes aegypti* and *Culex quinquefasciatus*. This simulated the activity of CO_2_. In related studies, acetone and cyclopentanone have also been tested as substitutes for CO_2_ [15–17] but with little success under field conditions [16, 17]. The current study sought to: (a) evaluate the synergistic importance of CO_2_ as a mosquito attractant in counter-flow MM-X traps; (b) assess the attraction of mosquitoes to different concentrations of 2-butanone; (c) determine the optimal concentration of 2-butanone for attracting mosquitoes; and, (d) evaluate the attraction of mosquitoes to odour baits containing 2-butanone in the field.

## Methods

### Mosquito rearing

All semi-field experiments reported in this article utilized laboratory colonies of the Mbita strains of *An. gambiae* or *Anopheles arabiensis*. The mosquitoes were reared under ambient environmental conditions at the Thomas Odhiambo Campus of the International Centre of Insect Physiology and Ecology (*icipe*-TOC) located near Mbita Point Township in western Kenya. Mosquito eggs were placed in plastic trays containing water from Lake Victoria. Water was filtered through charcoal to remove sediments. Larvae were fed on GO-CAT^®^ Complete cat food (Purina, Nestle S.A) supplied three times a day (0.03 mg/larva/day). Pupae were collected in clean cups daily. Collected pupae were transferred to an adult holding room and placed in mesh-covered cages (30 × 30 × 30 cm) prior to adult emergence. The adults were fed on 6% glucose solution through wicks made from absorbent tissue paper. The mosquitoes were randomly aspirated from the cages into paper cups using hand-held mouth aspirators and were starved for 8 h prior to experiments. The mosquitoes were only supplied with water from a wet cotton cloth material placed on top of holding cups during starvation.

### Synthetic mosquito attractants

The combination of CO_2_ and a synthetic mosquito attractant blend, referred to as the Mbita Blend 5 or MB5 [18] was used as a reference standard. The chemical constituents of MB5 included ammonia (2.5%), lactic acid (85%), tetradecanoic acid (0.00025%), 3-methyl-1-butanol (0.000001%), and 1-butylamine (0.001). All these chemical compounds were purchased from Sigma Aldrich Chemicals GmbH (Germany). Carbon dioxide was produced at the rate of 80.63 ± 2.82 ml/min by mixing 250 g of molasses (Mumias Sugar Company Ltd, Kenya), 17.5 g dry yeast (Angel^®^ Yeast Company Ltd, China) and 2 l of water [13]. Each component of MB5 was dispensed from individual strips (measuring 26.5 cm × 1.0 cm) of nylon stockings [19, 20]. The nylon strips (15 denier microfibres, 90% polyamide, 10% spandex) were purchased from Bata Shoe Company Ltd, Kenya.

### Semi-field experimental set up

**Fig. 1:**
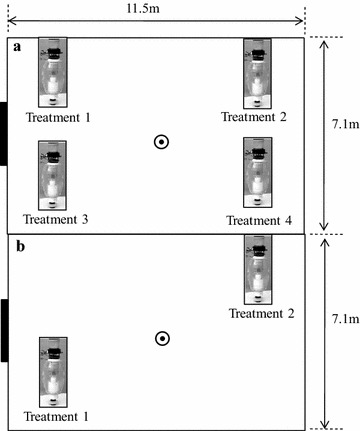
Four-choice (**a**) and two-choice (**b**) semi-field experimental set ups used to evaluate attraction of malaria mosquitoes to attractant blends containing 2-butanone in place of carbon dioxide. The position of the MMX-traps, the mosquito release point  and the entrance to the screen-house () are shown

All semi-field experiments were carried out inside screen-walled greenhouses at *icipe*-TOC (00°25′S, 34°13′E). The floor of the screen house (11.5 m × 7.3 m) was covered with sand, and watered daily to keep the microclimate humid and avert deaths of experimental mosquitoes due to desiccation. Each semi-field experiment was run between 20.00 and 06.30 h by utilizing 200 adult female mosquitoes. The mosquitoes, aged three to 6 days post-emergence and which had no prior access to a blood meal, were released at the centre of the screen-house in all replicates. MM-X traps containing four different test odours were placed at the four corners of the screen-walled greenhouse in four choice tests (Fig. 1a) or at two diagonal corners of the screen-house in two choice tests (Fig. 1b). The treatments were rotated in sequence until each had occupied every corner of the screen-house four times or two opposite corners of the greenhouse along a diagonal axis two times. The traps were rotated to eliminate any positional bias. During an experimental replicate the mosquitoes freely accessed all treatments, which were availed simultaneously in the screen-house enclosure on each experimental night. Mosquitoes attracted to specific treatments were caught in MM-X traps which functioned to both disperse the odours as well as capture attracted mosquitoes [21]. At the end of each experiment, trapped mosquitoes were taken to the laboratory, immobilized by freezing at −20°C and counted.

### Field study site

Field studies were conducted at Kigoche village near Ahero Town (00°08^1^S, 034°55^1^E) in Kisumu County, Western Kenya. Kigoche receives long rains from April to June and short rains from September to October yearly. The village lies at an altitude of 1160 m above sea level with an average relative humidity of 65% and an average annual rainfall range of 1000–1800 mm. Most residents in Kigoche engage in irrigated rice farming, which creates breeding sites for malaria mosquitoes. The houses are mainly covered with corrugated iron sheet roofs and have mud walls with open eaves through which mosquitoes enter [22]. *Anopheles arabiensis* and *Anopheles funestus* are the principal vectors of malaria in the area [23].

### Synergistic importance of carbon dioxide as a mosquito attractant

The primary aim of this baseline study was to examine the synergistic attraction of malaria mosquitoes to CO_2_ when augmented with other chemical compounds. This was achieved with a laboratory colony of *An. gambiae* mosquitoes using a fully replicated 4 × 4 Latin Square experimental design. The design included four treatments (Fig. 1): (a) no bait (the control); (b) the MB5 reference attractant blend plus CO_2_ (MB5 + CO_2_); (c) MB5 alone; and, (d) CO_2_ alone. The experiments were carried out for 16 nights.

### Attraction of malaria mosquitoes to different concentrations of 2-butanone

Semi-field behavioural experiments were carried out to determine the best concentration at which 2-butanone attracts mosquitoes. Dual-choice assays compared behavioural responses of *An. gambiae* mosquitoes towards a reference treatment (MB5 + CO_2_) versus a test treatment (i.e., MB5 augmented with various dilutions of 2-butanone in distilled water, namely 99.5, 10, 1.0, 0.1, 0.01, 0.004, 0.001, and 0.0004%). Thus, a total of eight dual-choice experiments were carried out. The different dilutions of 2-butanone in the test treatments acted as substitutes for CO_2_. Each dual-choice assay was carried out over a period of four nights (Fig. 1).

### The optimal concentration of 2-butanone that attracts malaria mosquitoes

The aim of this set of experiments was to determine if 2-butanone can mimic the synergistic effects of CO_2_ and, therefore, act as a substitute for this gas in synthetic attractants for mosquitoes. The experiments aimed to determine which of the two most promising concentrations of 2-butanone, (99.5 and 1% as determined in the previous experiments), served better as a replacement for CO_2_ in attractants for mosquitoes. The treatments included: (a) no bait (the control); (b) MB5 + CO_2_; (c) MB5 + 99.5% 2-butanone; and, (d) MB5 + 1.0% 2-butanone (Fig. 1). Female *An. gambiae* or *An. arabiensis* mosquitoes were released in separate screen houses on each experimental night for 16 nights each.

### Attraction of malaria mosquitoes to 2-butanone-based odour baits in the field

Indirect experimental comparisons were carried out indoors and outdoors in Kigoche village in western Kenya to evaluate the capacity of 2-butanone-based odour baits to attract mosquitoes under field conditions. In the indoor scenario, six houses each separated by a distance of ≥25 m were selected. All houses in the village were entered into an Excel spread sheet and computer-generated random numbers were used to select the six houses to be used in experiments. All selected houses measured between 15.0 and 20.0 sq m ground surface area. The six houses all had mud walls and floors with open eaves, corrugated iron-sheet roofs, no ceiling, and were either single or double roomed [24]. They were located on a transect oriented east–west along the northern edge of the Ahero rice irrigation scheme, approximately 28–150 m apart, 10–20 m away from cowsheds and within a range of 100 m from irrigation water channels and rice paddies [13, 25]. The selected houses had no occupants during experiments. Each house was assigned one of six treatments per night on a strict rotational basis to exclude positional bias. In the outdoor scenario, six open sites separated by a distance of ≥25 m were selected. The outdoor experimental sites had only short grass with no tall vegetation, were situated ≥25 m from the nearest house or cowshed and were located ≥100 m from the nearest mosquito larval breeding habitat. Each outdoor site was assigned one of six treatments per night on a rotational basis to exclude positional bias.

The six treatments which were allocated to the indoor and outdoor sites included: (a) no bait (i.e. the negative control); (b) CO_2_; (c) 99.5% 2-butanone; (d) MB5; (e) MB5 + CO_2_; and (f) MB5 + 99.5% 2-butanone. All chemical constituents of the odour baits, except CO_2_, were released using nylon strips [19, 23, 26]. All treatments were simultaneously dispensed using MM-X traps, which were hung 15 cm above the ground from a roof pole in indoor experiments and on a tripod stand in the outdoor scenario. The two studies were run from 18.00 to 06.00 h concurrently for 30 nights. All the traps were collected the following morning and transported to a field laboratory located at the Ahero Multipurpose Development Training Institute (AMDTI) where the mosquitoes were immobilized by freezing at −20 °C prior to counting. All female *Anopheles* mosquitoes were preserved in Eppendorf tubes containing 80% ethanol. Subsamples of collected mosquitoes belonging to the *An. gambiae* complex and the *An. funestus* group were identified to species level using molecular tools [27, 28].

### Ethical considerations

This study was approved by the ethical review committee of the Kenya Medical Research Institute (KEMRI/RES/7/3/1). The purpose and procedures of the study were explained to local leaders, household heads and resident volunteers in Kigoche village. The houses for the study were selected randomly and permission sought from the household head before experiments were rolled out.

### Data analysis

Mean mosquito catches were calculated in all experiments. The effect of a treatment as a major predictor of the number of mosquitoes caught in a trap under semi-field conditions was modelled using generalized linear models (GLM) with a Poisson distribution and a log link function. Data collected during field experiments were analysed using GLM fitted with negative binomial distribution and a log link function. The effects of treatments and house position on mosquito catches were tested as parameters in the model while night effect was captured as a covariate. All analyses were performed using IBM SPSS Statistics, version 20.0.

## Results

The work reported in this paper was carried out between August 2012 and August 2013. The semi-field experiments used a total of 16,000 female mosquitoes comprising 12,800 *An. gambiae* and 3200 *An. arabiensis*.

### Synergistic importance of carbon dioxide as a mosquito attractant

**Fig. 2:**
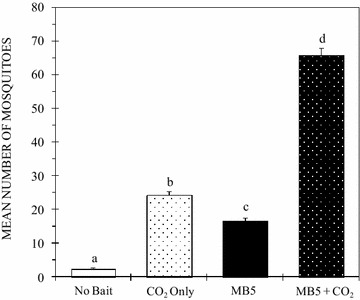
Mean numbers of *Anopheles gambiae* mosquitoes caught in an MM-X trap without a bait or in a trap baited with either CO_2_ alone, Mbita blend alone (MB5) or MB5 + CO_2_. *Error bars* denote the standard error of the mean number of mosquitoes trapped. *Bars* with *different letters* denote significant differences in the number of mosquitoes caught in traps containing each of the lures

The semi-field experiments carried out to measure the synergistic effect of CO_2_ as an ingredient in mosquito attractants were conducted over a period of 16 nights. Out of the 3200 female *An. gambiae* mosquitoes released, 1743 (54.5%) were recaptured. Mosquito catches differed significantly among the four treatments (P = 0.001). The MB5 reference attractant blend with CO_2_ (MB5 + CO_2_) attracted significantly more mosquitoes (n = 1053; mean = 65.81 ± 2.03) than MB5 alone (n = 264; mean = 16.5 ± 1.02; P = 0.001), CO_2_ alone (n = 388; mean = 24.25 ± 1.23; P = 0.001) and a trap with no bait (n = 38; mean = 2.38 ± 0.39; P = 0.001) (Fig. 2).

### Attraction of mosquitoes to different concentrations of 2-butanone

These experiments were carried out over a period of 32 nights. Of the 6400 female *An. gambiae* mosquitoes released, 4271 (66.7%) were recaptured. The mosquitoes trapped in each complete dual-choice comparison ranged from 51 to 77% of the 200 that were released in each replicate. In all cases the reference treatment (MB5 + CO_2_) used as a positive control, attracted a significantly more mosquitoes than the test treatments (P = 0.001 in all cases; Table 1). The pure form (99.5%) and the 1.0% dilution of 2-butanone were the most promising concentrations and were subsequently used to evaluate whether 2-butanone could substitute CO_2_ in a blend of synthetic mosquito attractants.

**Table 1:** Mean number (±SE) of *Anopheles gambiae* mosquitoes attracted to MM-X traps containing the reference (MB5 + CO_2_) and test treatments (MB5 + dilution ‘X’ of 2-butanone) of candidate synthetic mosquito attractant blends

Exp	Dilution of 2-butanone (‘X’), %	N	n	%Response	Mean (±SE) mosquito catches		Exp (B)
					Reference (MB5 + CO_2_)	Test treatment (MB5 + ‘X’)	
1	99.5	4	622	77.75	122.75 ± 5.54	32.75 ± 2.86	3.748*
2	10	4	559	69.88	122.75 ± 5.54	17.00 ± 2.06	7.221*
3	1.0	4	588	73.50	118.75 ± 5.45	28.25 ± 2.66	4.204*
4	0.1	4	526	65.75	115.00 ± 5.36	16.50 ± 2.03	6.970*
5	0.01	4	415	51.88	89.75 ± 4.74	14.00 ± 1.87	6.411*
6	0.004	4	543	67.88	118.50 ± 5.44	17.25 ± 2.08	6.870*
7	0.001	4	535	66.88	118.00 ± 5.43	15.75 ± 1.98	7.492*
8	0.0004	4	483	60.38	106.50 ± 5.16	14.25 ± 1.89	7.474*

The number of replicates (N), the number (n) and percentage (%) of trapped mosquitoes, test statistic {Exp(B)} and the level of statistical significance (* indicates P < 0.001) in each dual choice experiment is shown. 800 female *An. gambiae* were released across each series of four replicates

### The optimal concentration of 2-butanone that attracts mosquitoes

**Fig. 3:**
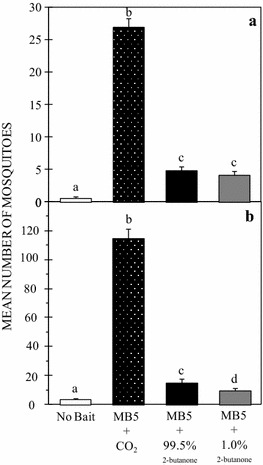
Mean number of *Anopheles arabiensis* (**a**) and *Anopheles gambiae* (**b**) mosquitoes caught in an MM-X trap with no bait, in a trap baited with either MB5 + CO_2_, MB5 + 99.5% 2-butanone or MB5 + 1.0% 2-butanone. *Error bars* denote the standard error of the mean number of mosquitoes trapped. *Bars* with *different letters* denote statistically significant differences in the number of mosquitoes trapped

Tests with *An. arabiensis* were conducted in May 2013. A total of 585 female *An. arabiensis* mosquitoes were recaptured out of the 3200 released. The mean numbers of mosquitoes that were caught in the trap with no bait, or the traps baited with MB5 + 1.0% 2-butanone, MB5 + 99.5% 2-butanone and MB5 + CO_2_ were 0.56 ± 0.19 (n = 9), 4.9 ± 0.51 (n = 67), 4.88 ± 0.55 (n = 78) and 26.94 ± 1.3 (n = 431), respectively (Fig. 3a). Whereas MB5 + CO_2_ attracted the majority of *An. arabiensis* (P = 0.001), the numbers of mosquitoes attracted to MB5 + 1.0% 2-butanone and MB5 + 99.5% 2-butanone did not differ significantly (P = 0.361).

Tests with *An. gambiae* were conducted in December 2012. For this species 2294 out of the 3200 mosquitoes released were recaptured. The mean numbers of *An. gambiae* mosquitoes caught in the trap with no bait, or the traps baited with MB5 + 1.0% 2-butanone, MB5 + 99.5% 2-butanone and MB5 + CO_2_ were 3.8 ± 0.49 (n = 61), 9.63 ± 0.78 (n = 154), 15.25 ± 0.98 (n = 244) and 114.69 ± 2.68 (n = 1835), respectively (Fig. 3b). The treatment containing MB5 + CO_2_ lured higher numbers of mosquitoes than all the other treatments (P = 0.001). A significantly higher number of the mosquitoes was attracted to the blend containing MB5 + 99.5% 2-butanone than that containing MB5 + 1.0% 2-butanone (P = 0.001). The blend with MB5 + 99.5% 2-butanone also attracted a significantly higher number of mosquitoes than the trap with no bait (P = 0.001).

### Attraction of malaria mosquitoes to 2-butanone based odour baits in the field

#### Indoor mosquito catches

**Fig. 4:**
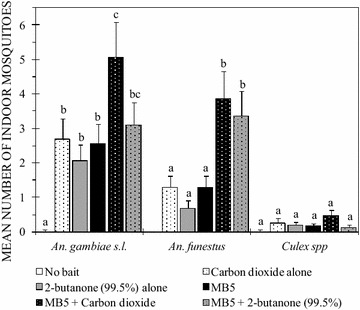
Mean number of wild female mosquitoes caught indoors in MM-X traps with no bait or in a trap baited with either CO_2_ alone, 2-butanone (99.5%) alone, MB5, MB5 + CO_2_, or MB5 + 2-butanone (99.5%). *Error bars* denote the standard error of the mean number of mosquitoes trapped. *Bars* with *different letters* within a given mosquito species denote significant differences in the number of mosquitoes trapped. No *An. funestus* were caught with the unbaited trap

All field studies were conducted during the dry season (from July to August 2013). The adult female mosquitoes collected indoors included *An. gambiae s.l.* (55.1%; n = 466), *An. funestus* (37.4%; n = 316), *Culex* spp (4.5%; n = 38), *Mansonia* spp (0.7%; n = 6) and other anopheline species (2.36%; n = 20) (Fig. 4). A total of 456 male adult mosquitoes comprising 76.1% *An. gambiae s.l.*, 19.3% *An. funestus*, 2.6%* Culex* and 2.0% *Mansonia* were collected indoors.

There was no significant difference between the attraction of female *An. gambiae s.l.* mosquitoes to MB5 + CO_2_ and MB5 + 99.5% 2-butanone (P = 0.090) (Fig. 4), and each of the two treatments was significantly more attractive to female *An. gambiae s.l.* compared to a trap with no bait (P = 0.001 for each). MB5 + CO_2_ attracted significantly more *An. gambiae s.l.* mosquitoes than either CO_2_ alone (P = 0.031), 99.5% 2-butanone alone (P = 0.003) or MB5 alone (P = 0.021). However, there was no difference between the capture rate of *An. gambiae s*.l. in traps containing MB5 + 99.5% 2-butanone compared with 99.5% 2-butanone alone, MB5 alone, or CO_2_ alone (P ≥ 0.05). The 466 *An. gambiae s.l.* mosquitoes trapped were 53.7% unfed; 12.2% blood-fed and 34.1% gravid.

Similarly, there was no difference in the response of female *An. funestus* mosquitoes to MB5 + CO_2_ or MB5 + 99.5% 2-butanone (P = 0.635), but each of the two treatments was significantly more attractive than any of the other blends (P = 0.001) (Fig. 4). The responses of *An. funestus* mosquitoes to CO_2_ alone, 2-butanone (99.5%) alone and MB5 alone were similar (P = 0.098). Of the 316 female *An. funestus* mosquitoes trapped, 97.2 were unfed, 0.3% were blood-fed and 2.5% were gravid.

#### Outdoor mosquito catches

**Fig. 5:**
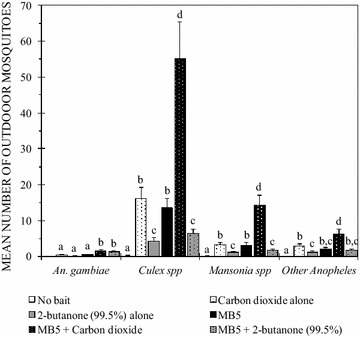
Mean number of wild female mosquitoes caught outdoors in MM-X traps with no bait or in a traps baited with either CO_2_ alone, 2-butanone (99.5%) alone, MB5, MB5 + CO_2_, or MB5 + 2-butanone (99.5%). *Error bars* denote the standard error of the mean number of mosquitoes trapped. *Bars* with *different letters* within a given mosquito species denote significant differences in the number of mosquitoes trapped. The numbers of *An. funestus* are not shown due to the very low catches

The adult female mosquitoes collected outdoors included *An. gambiae s.l.* (3%; n = 127), *An. funestus* (1.1%; n = 46), *Culex* (68.2%; n = 2889), *Mansonia* (17.2%; n = 730) and other *Anopheles* mosquito species (10.5%; n = 446) (Fig. 5). The 656 adult male mosquitoes trapped outdoors comprised 7.2% *An. gambiae s.l*., 1.7% *An. funestus, 76.2% Culex, 12.8% Mansonia* and 2.1% other *Anopheles* mosquito species.

There were no significant differences in the responses of female *An. gambiae s.l.* mosquitoes to MB5 + CO_2_ and MB5 + 99.5% 2-butanone (P = 0.544), but each of the two treatments was significantly more attractive than a trap with no bait (P = 0.001 for both). There were fewer *An. gambiae s.l*. mosquitoes collected in a trap baited with MB5 alone than MB5 + CO_2_ (P = 0.004) or MB5 + 99.5% of 2-butanone (P = 0.02). The responses of *An. gambiae s.l.* to CO_2_ alone were similar to 99.5% of 2-butanone alone (P = 0.147) and MB5 alone (0.884) but lower than MB5 + CO_2_ (P = 0.003) or MB5 + 99.5% of 2-butanone (P = 0.014). The female *An. gambiae s.l.* mosquitoes trapped were either unfed (83.5%; n = 106), blood fed (4.7%; n = 6) or gravid (11.8%; n = 15).

The response of female *An. funestus* mosquitoes to MB5 + CO_2_ and MB5 + 99.5% 2-butanone did not differ (P = 0.533). Likewise, there were no significant differences between the responses of *An. funestus* mosquitoes to CO_2_ alone and 2-butanone (99.5%) alone (P = 1.000). All the female *An. funestus* mosquitoes trapped were unfed.

### Identity of female *An. gambiae s.l.* and *An. funestus* mosquitoes by PCR

The 240 specimens of *An. gambiae s.l.* that were randomly selected from the 593 collected in total and analysed by PCR indicated the presence of 94.2% *An. arabiensis* and 5.8% *An. gambiae s.s.* Of the 105 random samples of *An. funestus* analysed out of the 362 collected in total, 97.3% were *An. funestus s.s.* while 2.7% could not be identified even after conducting repeated runs.

## Discussion

In this study, it was observed that the responses of laboratory-reared *An. gambiae s.s*. mosquitoes to the MB5 reference attractant blend with CO_2_ were significantly higher compared to MB5 alone, CO_2_ alone or a trap without a bait. In all semi-field investigations MB5 + CO_2_ attracted a significantly higher number of mosquitoes than its variants containing the different dilutions of 2-butanone used to replace CO_2_. When using the blends of MB5 + 2-butanone, the highest catches were associated with the 99.5% concentration of 2-butanone and the 1.0% concentration of 2-butanone. Overall catches of *An. arabiensis* were far much lower than those of *An. gambiae* under semi-field conditions, probably because the human-mimicking attractant blends were developed and customized using *An. gambiae* as the test organism [25, 26] and that *An. arabiensis* has a more opportunistic host preference [1]. In the field study, *An. gambiae s.l*., *An. funestus* and *Culex* species were attracted to both MB5 + CO2 and MB5 + 99.5% 2-butanone with no significant difference in catch size between the blends. These results demonstrate that 2-butanone can be used as a replacement for CO2 under field conditions.

The finding that significantly more laboratory-reared mosquitoes were attracted to MB5 + CO_2_ than to MB5 alone (P < 0.001), CO_2_ alone (P < 0.001) or a trap without a bait (P < 0.001) underscores the action of CO_2_ as a synergist in mosquito attractants [21, 29]. The gas is known to activate mosquitoes by eliciting take-off behaviour and sustaining them in host-seeking flight [6, 7, 30]. These findings are in line with the results of studies which demonstrated that compounds are more attractive to host-seeking mosquitoes when blended than when applied alone [31].

It was observed that the pure (99.5%) form of 2-butanone is a potential replacement for CO_2_ in mosquito attractants. Under field conditions there were no differences between the numbers of *An. gambiae s.l.* and *An. funestus* mosquitoes attracted to MB5 + CO_2_ compared with MB5 + 99.5% 2-butanone. 2-butanone is a natural product identified in the emanations of various vertebrates and arthropods [32, 33] and several insects express a behavioural response upon exposure to this compound. Two separate studies [34, 35] have reported that the olfactory receptor cell of the fruit fly *Bactrocera tyoni* that responds to CO_2_ also responds to 2-butanone. And more recently, Turner et al. [14] demonstrated the capacity of 2-butanone to induce a dose-dependent activation of the CO_2_ receptor neuron in the maxillary palps of *An. gambiae, Aedes aegypti* and *Culex quinquefasciatus*. Failure to observe this effect under semi-field conditions may imply that 2-butanone acts as a long range rather than a short or medium-range cue or that the proximity of a more attractive alternative (MB5 + CO_2_) was preferred when mosquitoes were presented with a direct choice. Furthermore, there were no statistical differences in the numbers of *An. funestus* attracted to MB5 + CO_2_ or MB5 + 2-butanone (99.5%) under field conditions, but because a colony of this mosquito species has not been established at the research station in Mbita the response of this species under semi-field conditions could not be tested.

The relatively high number of wild male *An. gambiae s.l.* and *An. funestus* mosquitoes in traps baited with synthetic odour blends is contrary to expectations because males are phytophagous and are thought unlikely to respond to host-seeking odour blends compared to female mosquitoes [3, 37]. It may be assumed that the males were pursuing the females for mating, if this life history trait ever occurs indoors without swarming. Currently, there is an urgent need for potent synthetic odour blends for sampling and control of male mosquitoes. Such blends could be deployed to reduce mating success, and also to reduce the number of gravid female mosquitoes and prevalence of mosquito-borne diseases. Because the prospects of eliminating malaria are largely threatened by rapid development of drug-resistant *Plasmodium* parasites and insecticide-resistant malaria vectors, novel tools are needed. Odour-baited trapping technology has been used successfully in western Kenya to reduce mosquito bites and malaria prevalence [38]. The number of mosquitoes around houses was reduced by mass deployment of outdoor traps that were baited with MB5 augmented with 2-butanone instead of CO_2_ [36, 38]. The baited traps were effectively and repeatedly used for removal trapping of outdoor-biting mosquito vectors. Although residual attraction of mosquitoes to synthetic compounds impregnated on nylon strips has been reported [23], similar studies are needed for mosquito attractants that are augmented with 2-butanone. An odour bait with a residual activity of long duration without the need for frequent replenishment would be convenient for both monitoring as well as removal trapping of mosquitoes in remote areas of sub-Saharan Africa.

Human landing catches, light traps, bed nets occupied by humans, pyrethrum spray catches, and man-landing catches are commonly used for sampling of malaria vectors and estimation of malaria transmission intensity [39]. The methods vary in terms of reliability and efficacy, hence the need for standardized tools that are sensitive, specific, reliable and ethically acceptable for trapping and sampling malaria vectors. Recent studies indicate that synthetic odour baits dispensed by MM-X traps can be used reliably to collect live and species specific samples of both indoor and outdoor biting malaria and other mosquito vectors, particularly those which are host-seeking [13, 18, 29]. Thus, it is important to compare the odour blend, putative CO_2_ replacement and odour baited traps that were used in the current study with the common trapping tools outlined above.

## Conclusion

This study demonstrates that 2-butanone has the potential to serve as a substitute for carbon dioxide in synthetic mosquito attractants, which is an essential step towards the development of sustainable, olfactory-based tools for mosquito vector control and surveillance [36]. The study further emphasizes the possibility of using OBTs for monitoring, surveillance and control of malaria and other mosquito vectors. Further studies are needed to evaluate the residual activity of 2-butanone in lures for mosquitoes as well as to understand more about its mode of action.
